# Molecular characterization of immunoinhibitory factors PD-1/PD-L1 in chickens infected with Marek’s disease virus

**DOI:** 10.1186/1743-422X-9-94

**Published:** 2012-05-21

**Authors:** Ayumi Matsuyama-Kato, Shiro Murata, Masayoshi Isezaki, Rika Kano, Sara Takasaki, Osamu Ichii, Satoru Konnai, Kazuhiko Ohashi

**Affiliations:** 1 Department of Disease Control, Graduate School of Veterinary Medicine, Hokkaido University, Kita-18, Nishi-9, Kita-ku, Sapporo, 060-0818, Japan; 2 Department of Biomedical Sciences, Graduate School of Veterinary Medicine, Hokkaido University, Kita-18, Nishi-9, Kita-ku, Sapporo, 060-0818, Japan

## Abstract

**Background:**

An immunoinhibitory receptor, programmed death-1 (PD-1), and its ligand, programmed death-ligand 1 (PD-L1), are involved in immune evasion mechanisms for several pathogens causing chronic infections and for neoplastic diseases. However, little has been reported for the functions of these molecules in chickens. Thus, in this study, their expressions and roles were analyzed in chickens infected with Marek’s disease virus (MDV), which induces immunosuppression in infected chickens.

**Results:**

A chicken T cell line, Lee1, which constitutively produces IFN-γ was co-cultured with DF-1 cells, which is a spontaneously immortalized chicken fibroblast cell line, transiently expressing PD-L1, and the *IFN-γ* expression level was analyzed in the cell line by real-time RT-PCR. The *IFN-γ* expression was significantly decreased in Lee1 cells co-cultured with DF-1 cells expressing PD-L1. The expression level of *PD-1* was increased in chickens at the early cytolytic phase of the MDV infection, while the *PD-L1* expression level was increased at the latent phase. In addition, the expression levels of *PD-1* and *PD-L1* were increased at tumor lesions found in MDV-challenged chickens. The expressions levels of *PD-1* and *PD-L1* were also increased in the spleens and tumors derived from MDV-infected chickens in the field.

**Conclusions:**

We demonstrated that the chicken PD-1/PD-L1 pathway has immunoinhibitory functions, and PD-1 may be involved in MD pathogenesis at the early cytolytic phase of the MDV infection, whereas PD-L1 could contribute to the establishment and maintenance of MDV latency. We also observed the increased expressions of *PD-1* and *PD-L1* in tumors from MDV-infected chickens, suggesting that tumor cells transformed by MDV highly express PD-1 and PD-L1 and thereby could evade from immune responses of the host.

## Background

Marek’s disease (MD) is a viral lymphoproliferative disease of chickens caused by a cell-associated herpesvirus, Marek’s disease virus (MDV; family *Herpesviridae*, subfamily *Alphaherpesvirinae*, genus *Mardivirus*, species *Gallid Herpesvirus* 2 (GaHV-2)) [1]. MDV strains are classified into 3 serotypes, GaHV-2 (MDV serotype 1: MDV-1), *Gallid herpesvirus* 3 (MDV serotype 2: MDV-2) and *Meleagrid herpesvirus* 1 (MDV serotype 3 or herpesviru of turkeys (HVT)), and MD is caused by serotype1 MDV strains except for attenuated vaccine strains [1]. The pathogenesis of MD can be sequentially divided into 3 phases: early cytolytic phase, latent phase, and secondary cytolytic phase with immunosuppression and tumor development. In the early cytolytic phase, MDV-1 causes lytic infection of lymphoid cells, mainly B cells that last for up to six days after infection [2]. Then, this cytolytic infection induces the activation of T cells, and MDV establishes latency in a part of the activated CD4^+^ T cells at 1–2 weeks after infection. In the latent phase, infected chickens show no clinical signs, but cellular immunity is continually inhibited by apoptosis of CD4^+^ T cells, CD8-down regulation in CD8^+^ T cells, decrease in the responsiveness to the stimulation through T cell receptor (TCR) in CD4^+^ and CD8^+^ T cells and MHC class I-down regulation at 2–3 weeks after infection [3,4]. In the secondary cytolytic phase, MDV-1 transforms a few latently infected CD4^+^ T cells, and develops malignant lymphomas. The main targets for the transformation by MDV-1 are CD4^+^ T cells, suggesting that latent infection in this T cell subset is intimately related to the subsequent transformation by MDV-1 [1]. Several viral factors which could contribute to the oncogenicity and pathogenicity of MDV-1 have been identified. Among them, a viral protein, Meq, is the most important factor involved in MDV-1 oncogenicity [5-8]. Meq is only present in MDV-1 strains and is abundantly expressed in MDV-1-transformed cell lines and tumor samples. The overexpression of Meq in rodent and chicken fibroblast cell lines resulted in morphological changes of the cells and protection of the cells from apoptosis [6,7]. A Meq-deficient mutant virus was completely non-oncogenic [8]. These observations suggest that Meq plays a key role in the oncogenicity of MDV-1. However, the precise molecular mechanism of MDV-1 oncogenicity and pathogenicity remains to be established due to the lack of appropriate T cell transformation systems in chickens.

Currently, MD is well controlled by the vaccination with MDV-1 strains, apathogenic MDV-2 strains and/or HVT. However, MDV-1 strains in the field tend to increase their virulence, and increased numbers of MD cases have been reported even in vaccinated chickens [9]. Therefore, the development of more effective vaccines would be desirable, since future outbreaks of MDV-1 could occur [9]. However, the detailed mechanisms for the protection by vaccines are still unknown. It has been known that cell-mediated immunity is effective for the inhibition of MDV-1 propagation and oncogenicity [10]. The immune responses induced by MDV-1 inhibit virus propagation, whereas an excess of inflammatory response induces immunosuppression through a negative feedback mechanism, and subsequently contributes to MDV-1 reactivation from latency [11]. Thus, the host immune responses in infected chickens are also involved in the pathogenesis of MDV-1.

Persistent viral infections often result in T cell exhaustion. During chronic viral infection, such as human immunodeficiency virus (HIV) and hepatitis C virus (HCV), antigen-specific CD8^+^ T cells initially obtain functional activities but gradually become dysfunctional as the infection progresses, and exhausted CD8^+^ T cells are unable to produce sufficient cytokines [12,13]. An immune inhibitory receptor, programmed death 1 (PD-1) and its ligand, programmed death ligand 1 (PD-L1), have been reported as molecules involved in T cell exhaustion [14]. PD-1 and PD-L1 belong to the B7-CD28 superfamily, and PD-1 is expressed on the membrane of activated T cells and B cells, while PD-L1 is constitutively expressed on the membrane of activated T cells, dendritic cells (DCs), macrophages, and a wide range of non-hematopoietic cells [15,16]. PD-1 expression is upregulated on CD8^+^ T cells specific for cells chronically infected with human T-cell lymphotropic virus type 1 (HTLV-1) [17]. The increase in PD-L1 expression was observed in cells infected with hepatitis B virus, HIV, and HTLV-1 [17-19]. In addition, the PD-1/ PD-L1 pathway plays a crucial role in immune evasion by tumor cells, such as pancreatic cancer and adult T-cell leukemia [15,16].

Chicken PD-1 and PD-L1 shared 58% and 54.5% amino acid identities with human PD-1 and PD-L1, respectively, and the chicken PD-1/PD-L1 pathway may also play roles in the immunoinhibitory functions. Recently, it has been reported that the expression levels of *PD-1* and *PD-L2* mRNA on CD4^+^ T cells were increased in the secondary cytolytic phase of the MDV-1 infection [20]. PD-L2, as well as PD-L1, belongs to the B7-CD28 superfamily, and is known to carry the immunoinhibitory functions by the interaction with PD-1 [21]. However, the PD-1/PD-L1 pathway usually serves as an immunoinhibitory molecule in human and murine diseases such as chronic infections and tumors, but not PD-L2. Therefore, it is necessary to analyze the expressions of PD-1 and PD-L1 in MDV-1-infected chickens and MD-derived tumors to properly know the roles of chicken immunoinhibitory molecules in MD pathogenesis and tumorigenesis. In this study, we evaluated the immunosuppressive functions of the PD-1/PD-L1 pathway, and analyzed the expression kinetics of the immunoinhibitory molecules in the spleens, peripheral blood mononuclear cells (PBMCs), and tumors of MDV-infected chickens. These results suggest that the chicken PD-1/PD-L1 pathway has immunosuppressive functions, and PD-1 and PD-L1 may contribute to MD pathogenesis and tumorigenesis.

## Results

### Evaluation of the immunoinhibitory function of the PD-1/PD-L1 pathway

**Figure 1:**
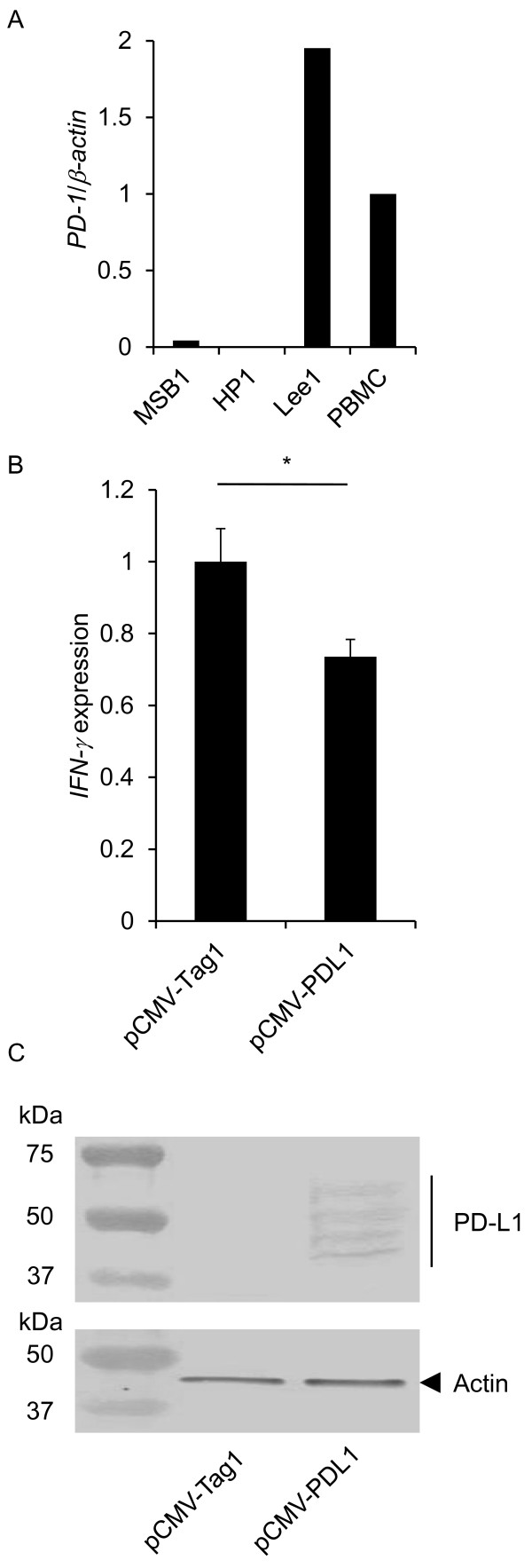
**Analysis of immunoinhibitory functions by chicken PD-1/PD-L pathway.**(**A**) The expression of *PD-1* mRNA was determined in MSB1, HP1, Lee1 cell, and PBMCs from uninfected chickens by real-time RT-PCR. The degree of the *PD-1* mRNA expression was expressed relative to the mean basal value in PBMCs after normalization to that of *β-actin* mRNA. (**B**) The expression levels of *IFN-γ* mRNA in Lee1 cells co-cultured with DF-1 cells were determined by real-time RT-PCR. DF-1 cells were transfected with pCMV-Tag1 or pCMV-PDL1. The degree of the *PD-1* and *IFN-γ* mRNA expression was expressed relative to the mean basal value in the presence of pCMV-Tag1 after normalization to that of *β-actin* mRNA. Three independent experiments were performed in triplicate. Error bars indicate standard deviations. The significant differences were determined by Student’s paired *t*-test between types of expression plasmids (*P < 0.05). (**C**) Transient expression of PD-L1 construct in DF-1 cells was determined by western blotting. Blots were also probed with an anti-actin monoclonal antibody as a loading control.

In order to clarify the immunoinhibitory functions of chicken PD-1 and PD-L1, the expression level of *IFN-γ* mRNA was measured in a chicken T cell line co-cultured with cells expressing PD-L1. First, we measured the level of *PD-1* mRNA expression in three chicken T cell lines, MSB1 and HP1, which were transformed by MDV-1, and Lee1, which was transformed by reticuloendotheliosis virus (REV), and PBMCs obtained from a healthy chicken (Figure 1A). *PD-1* mRNA was highly expressed in Lee1 cells compared to other cells, and thus, Lee1 cells were used to evaluate the immunosuppressive functions of PD-1 and PD-L1. In this assay, we quantified the expression of *IFN-γ* mRNA in Lee1 cells after the co-culture with a chicken fibroblast cell line, DF-1, transfected with PD-L1-expressing vector, pCMV-PDL1 (Figure 1B), since REV-transformed T cells are known to constitutively produce IFN-γ [22]. The expression of IFN-γ mRNA was reduced in Lee1 cells co-cultured with pCMV-PDL1-transfected DF-1 cells compared to pCMV-Tag1-transfected DF-1 cells. The transient expression of PD-L1 in pCMV-PDL1-transfected DF-1 cells was confirmed by Western blotting (Figure 1C). These results suggest that the PD-1/PD-L1 pathway exhibits the immunosuppressive functions.

### MDV loads and the expressions of *PD-1*, *PD-L1*, *IFNγ*, and *meq* mRNA in experimentally infected chickens

**Figure 2:**
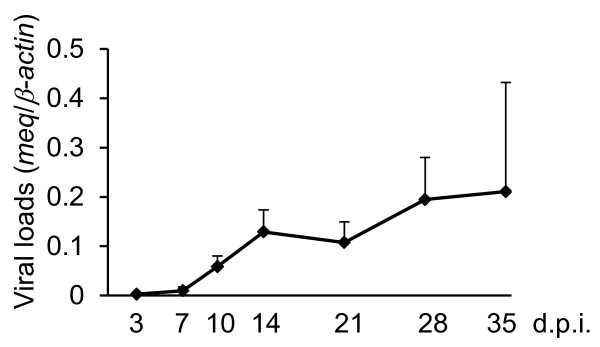
**Viral loads in spleens from chickens experimentally infected with MDV-1.** Chickens were inoculated with 2,000 PFU of RB1B (*n* = 36). Spleens were collected from MDV-1-infected and uninfected chickens at 3, 7, 10, 14, 21, 28, and 35 d.p.i., and four samples were prepared at each time point. Viral loads in the spleens were determined by real-time PCR targeting the *meq* gene. The results are shown as ratios between concentrations of the *meq* and chicken *β-actin* genes in the spleens. Error bars indicate standard deviations.

**Figure 3:**
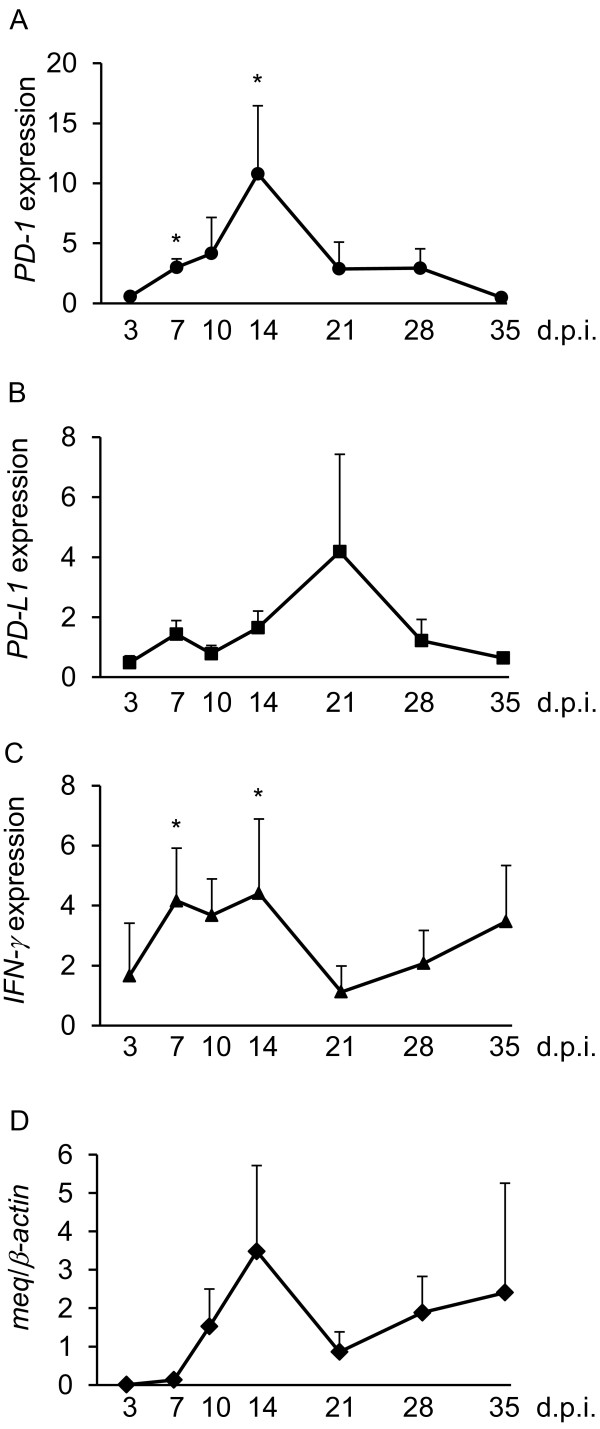
**The mRNA expressions of*PD-1*and*PD-L1*in the spleens from chickens experimentally infected with MDV-1.** The expressions of *PD-1* (**A**), *PD-L1* (**B**), *IFN-γ* (**C**), and *meq* (**D**) mRNA were determined by real-time RT-PCR. The results are presented as mean and standard deviations in each schedule. The concentration of each mRNA (**A-C**) was normalized to that of *β-actin* mRNA. The extent of gene expression (−fold) was calculated by dividing the value of each sample by that of each control. The significant differences were determined by Student’s *t*-test (*P < 0.05).

To investigate whether immunoinhibitory molecules are involved in MD pathogenesis, we analyzed viral loads and expression levels of host and viral genes in the spleens of MDV-1-infected chickens. First, the MDV-1 genome loads were quantified at 3, 7, 10, 14, 21, 28, and 35 day post inoculation (d.p.i) (Figure 2). The MDV-1 genome was detected in all of samples from infected chickens throughout the experimental period. The viral load was increased at 14 d.p.i., decreased slightly at 21 d.p.i., and increased again at 28 d.p.i. In our experiments, based on the amounts of the MDV-1 genome at each time point after virus inoculation, we determined each phase of the MDV infection: the early cytolytic phase (7–14 d.p.i.), the latent phase (14–28 d.p.i.), and the secondary cytolytic phase (after 28 d.p.i.). The expression of *PD-1* mRNA was upregulated in the early cytolytic phase, whereas the expression of *PD-L1* mRNA was transiently increased in the early cytolytic phase (at 7 d.p.i.) and increased again in the latent phase (at 21 d.p.i.) (Figure 3A, B). Since IFN-γ could be involved in the expressions of PD-1 and PD-L1 [14,15], the expression of *IFN-γ* mRNA was also analyzed (Figure 3C). The expression of *IFN-γ* mRNA was increased in the early infection phase (at 7–14 d.p.i.), decreased in the latent phase following the increase in the expression of *PD-L1* mRNA, and increased again in the secondary cytolytic phase.

In recent studies, it was reported that the HIV accessory protein Nef or the HCV core protein could upregulate PD-1 expression [23,24]. Since an MDV-1 oncoprotein, Meq, contributes to oncogenicity by altering the expression of various cellular genes [6] and also plays an important role in the induction of immunosuppression [25], Meq may be involved in the expression of PD-1 in MDV-1-infected chicken. In addition, Meq is abundantly expressed in latently-infected cells and tumor cells [5], and therefore, Meq may be also involved in the expression of PD-L1. Thus, we analyzed the expression of *meq* mRNA in each phase of the infection (Figure 3D). The expression of *meq* mRNA was increased at 14 d.p.i., and this expression kinetics were similar to those of *PD-1* mRNA in the early cytolytic phase. However, the expression of *meq* mRNA was increased again in the secondary cytolytic phase, unlike the kinetics of *PD-1* mRNA, because MDV-1 was reactivated following the disease progression (Figure 2).

### Expressions of *PD-1*, *PD-L1*, *IFNγ*, and *meq* mRNA in tumor lesions

**Figure 4:**
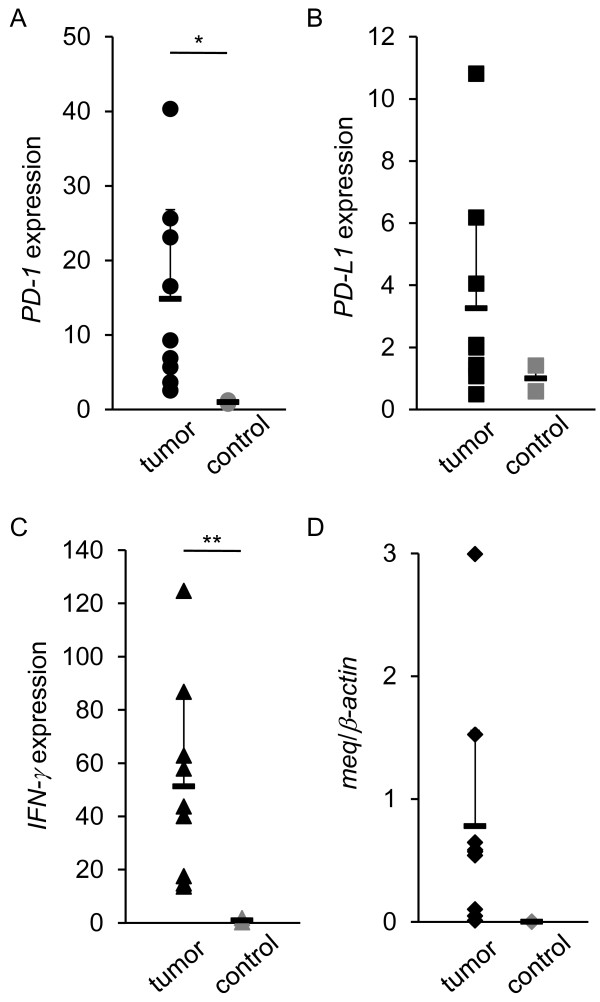
**The mRNA expressions of*PD-1*and*PD-L1*in tumor lesions observed in kidneys of chickens experimentally infected with MDV-1.** A total of 9 tumor samples were collected at 21, 28, and 35 d.p.i. (*n* = 2, 3, and 4, respectively). Two kidney samples were also collected from uninfected chickens and analyzed as controls. The expressions of *PD-1* (**A**), *PD-L1* (**B**), *IFN-γ* (**C**), and *meq* (**D**) mRNA were determined by real-time RT-PCR. The concentration of each mRNA was normalized to that of *β-actin* mRNA. The extent of gene expression (−fold) was calculated by dividing the value of each sample by that of the value of each control (**A-D**). Error bars represent standard deviations. The significant differences were determined by Student’s *t*-test (**P < 0.01).

**Figure 5:**
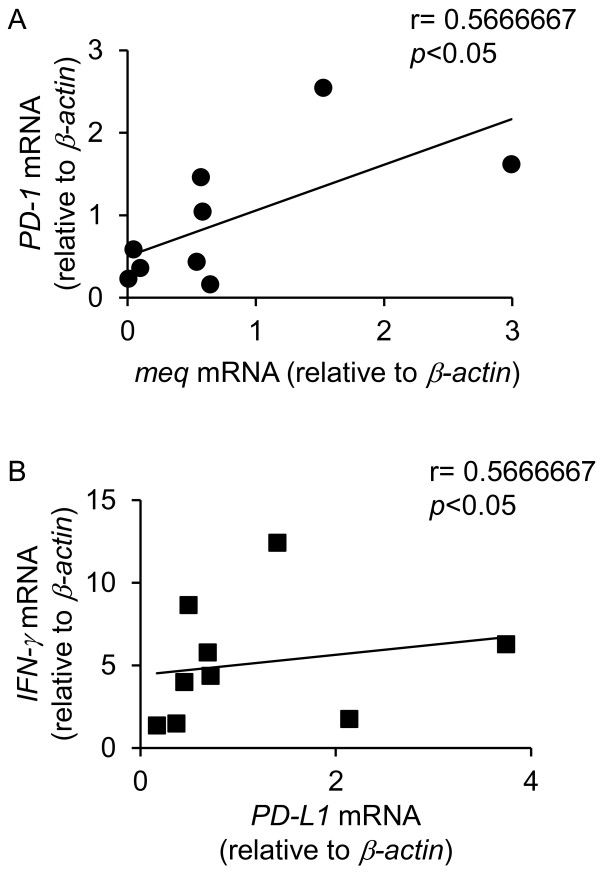
**A positive correlation between each of the gene expressions in MD-derived tumor samples.** These data are shown correlations between gene expressions of *meq* and *PD-1* (**A**), and *PD-L1* and *IFN-γ* (**B**). r, Spearman’s correlation coefficient.

To examine if immunoinhibitory molecules could be involved in tumor formation by MDV-1, the mRNA expressions of *PD-1*, *PD-L1*, *IFN-γ*, and *meq* were analyzed in tumor lesions observed in kidneys of experimentally infected chickens (Figure 4). The expressions of *PD-1* and *IFN-γ* in tumor lesions were significantly increased, and the mean degree of the expression of *PD-L1* was increased although the difference was not statistically significant. In addition, the expressions of *PD-1* and *PD-L1* in tumor lesions were compared with those in PBMCs obtained from experimentally infected chickens (Additional file 1: Figure S1). The expressions of *PD-1* and *PD-L1* in tumor lesions were significantly increased compared to those in PBMCs. These results suggest that PD-1 and PD-L1 were expressed on tumor cells caused by MDV-1. In tumor cells, there was a positive correlation between *meq* and *PD-1* mRNA expressions (Figure 5A), or between *PD-L1* and *IFN-γ* mRNA expressions (Figure 5B). However, no correlation was shown between the MDV-1 genome and the expressions of PD-1 or PD-L1. The Spearman correlation coefficient between the MDV-1 genome and PD-1 or PD-L1 was 0.18 (*p* = 0.64) and 0.050 (*p* = 0.90), respectively.

### Expressions of *PD-1*, *PD-L1*, *IFNγ*, and *meq* mRNA in the spleens and tumor lesions derived from chickens with MD in the field

**Figure 6:**
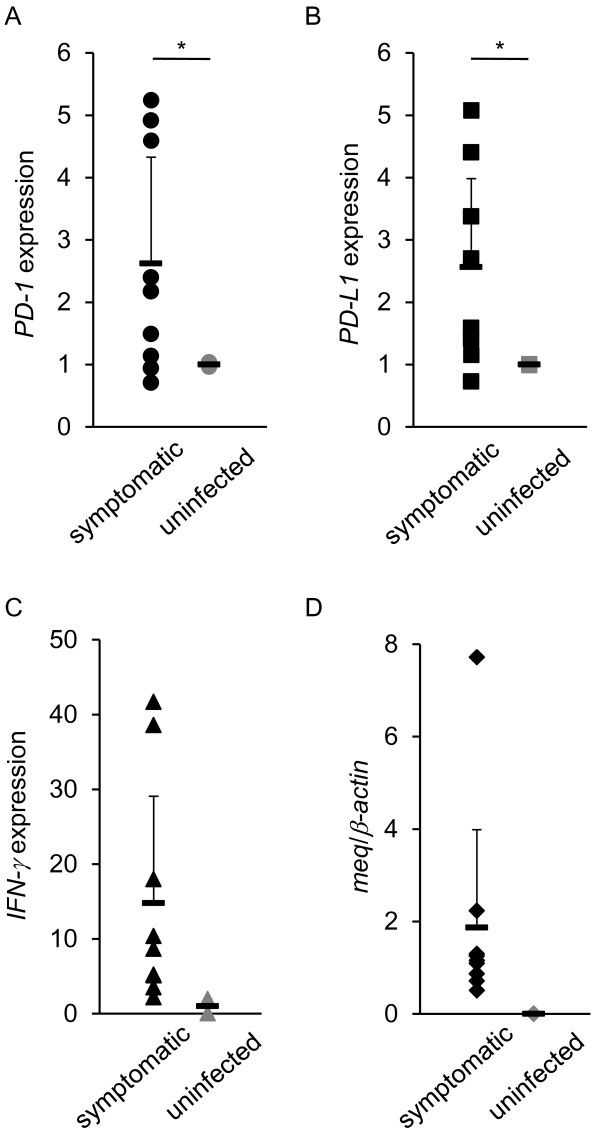
**The mRNA expressions of*PD-1*and*PD-L1*in the spleens of chickens with MD in the field.** A total of 9 spleen samples were collected from poultry farms. Two spleen samples were collected from uninfected chickens and analyzed as controls. The expressions of *PD-1* (**A**), *PD-L1* (**B**), *IFN-γ* (**C**), and *meq* (**D**) mRNA were determined by real-time RT-PCR. The concentration of each mRNA was normalized to that of *β-actin* mRNA. The extent of gene expression (−fold) was calculated by dividing the value of each sample by that of the value of each control (**A-D**). Error bars represent standard deviations. The significant differences were determined by Student’s *t*-test (*P < 0.05).

**Figure 7:**
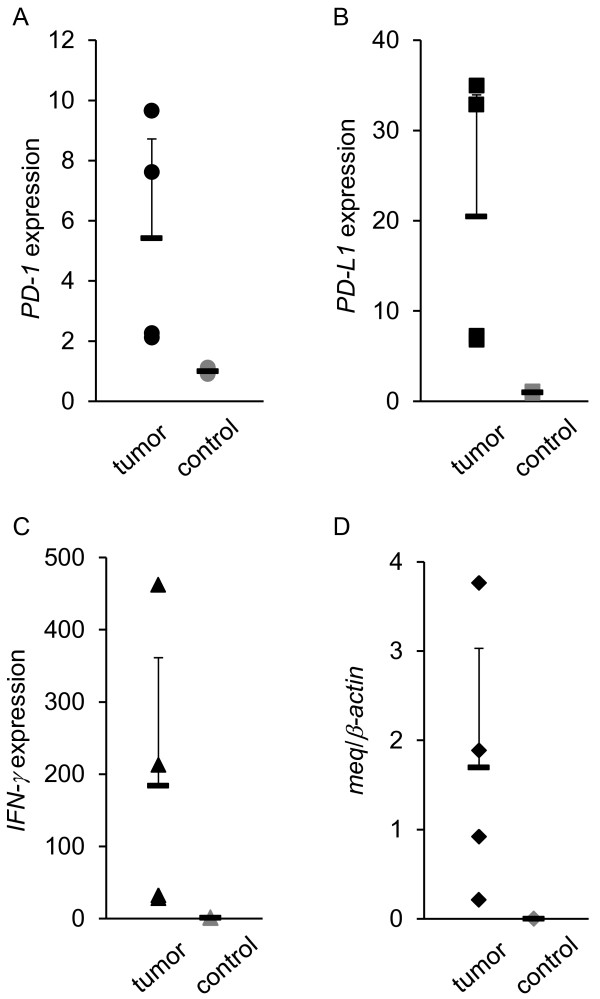
**The mRNA expressions of*PD-1*and*PD-L1*in tumor samples of chickens with MD in the field.** A total of 4 tumor samples were analyzed, and then, PBMCs from uninfected chickens (*n* = 3) were also analyzed as controls. The expressions of *PD-1* (**A**), *PD-L1* (**B**), *IFN-γ* (**C**), and *meq* (**D**) mRNA were determined by real-time RT-PCR. The concentration of each mRNA was normalized to that of *β-actin* mRNA. The extent of gene expression (−fold) was calculated by dividing the value of each sample by that of the value of each control (**A-D**). Error bars represent standard deviations.

To examine if immunoinhibitory molecules are expressed in chickens developing MD in the field as observed in those of experimentally infected chickens, we measured the mRNA expressions of the *meq* gene and host genes in the spleens and tumor lesions derived from chickens with MD in the field (Figures 6 and 7). The expression of *meq* RNA was observed in all of the field samples, and the mean degree of the mRNA expressions of *PD-1*, *PD-L1* and *IFN-γ* was also increased. Thus, in the case of chickens with MD in the field, the mRNA expressions of *PD-1* and *PD-L1* were similar to those observed in experimentally infected chickens.

### *in situ* detection of *PD-1*, *PD-L1*, and *meq* mRNA in tumors derived from MDV-infected chickens

**Figure 8:**
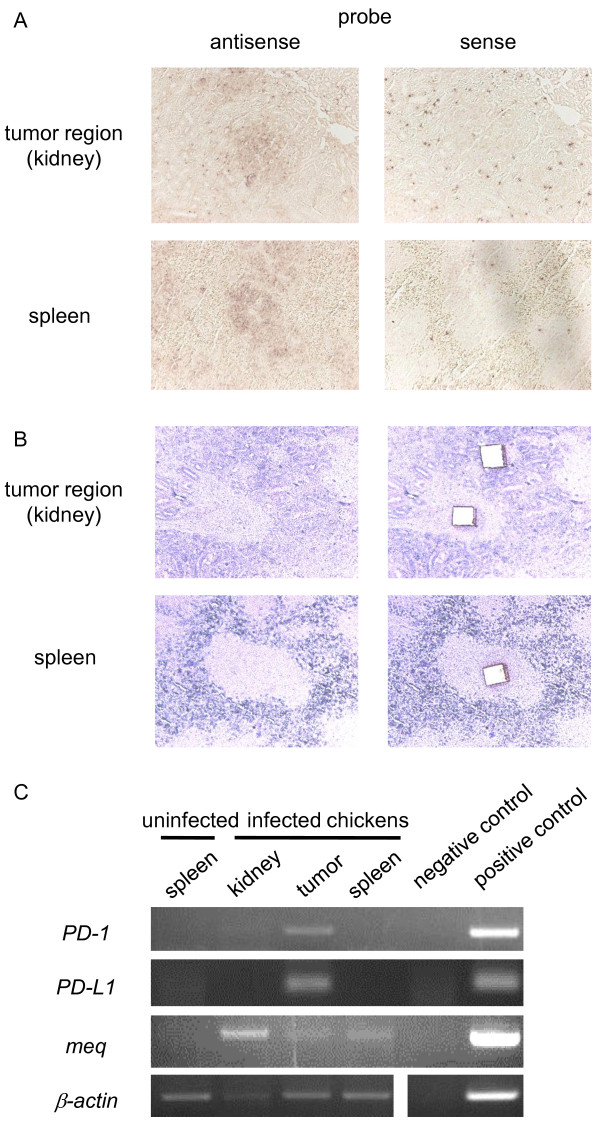
**The mRNA expressions of*PD-1*,*PD-L1*, and*meq*in MD-derived tumor cells.** (**A**) The expression of *meq* mRNA was confirmed in serial sections of spleen and tumor samples from experimentally infected chickens by *in situ* hybridization. Samples were collected at 35 d.p.i. Positive signals were detected mononuclear cells infiltrating into the renal tubulointerstitium (upper panel) and in white pulp of spleen (lower panel). (**B**) Mononuclear cells infiltrating into the renal tubulointerstitium and adjacent renal cells, and white pulp of the spleens from infected or uninfected chickens were captured by LMD. Panels show the targeted areas before (left) and after (right) capture. (**C**) The expression of *PD-1*, *PD-L1*, and *meq* mRNA was confirmed by RT-PCR using RNA isolated from LMD-captured samples.

To further determine if these host genes and the *meq* gene were expressed in MD tumor cells, the histological analysis was conducted by *in situ* hybridization. Since the *meq* gene is abundantly expressed even in latently-infected and transformed cells, it seems to be an optimal marker for the detection of MDV-infected cells. In tumor lesions, the *meq* transcripts were detected in cells infiltrating into the interstitial of kidney tubule, and in the spleen, *meq* transcripts were detected in white pulp (Figure 8A). These results indicate that MDV-1-infected cells could be detected by *in situ* hybridization analysis. However, the *PD-L1* transcripts were undetectable in the spleen and tumor lesions by *in situ* hybridization analysis (data not shown). In order to confirm the mRNA expressions of *PD-1* and *PD-L1* in tumor cells, nested RT-PCR assays were performed by using laser-captured microdissections (Figure 8B, C). The mRNA expressions of *PD-1* and *PD-L1* were observed in tumor cells. These results showed that both *PD-1* and *PD-L1* mRNA were expressed in MD-derived tumor cells, and may contribute to immune evasion of tumor cells from host immune responses.

## Discussion

Recent studies have shown that host immunoinhibitory factors, such as PD-1 and PD-L1, are exploited to evade the immune response in chronic viral infections [26,27]. However, the function of the chicken PD-1/PD-L1 pathway has not been well characterized. In order to evaluate immunosuppressive function of PD-1 / PD-L1 pathway, we determined the expression level of *IFN-γ* mRNA in Lee1 cells which was co-cultured with DF-1 expressing PD-L1. The expression of *IFN-γ* mRNA was decreased in Lee1 cells co-cultured with DF-1 expressing PD-L1, suggesting that in case of chickens, PD-L1 also interacts with PD-1, and then induces the immunosuppression in PD-1-expressing cells, although further functional analysis concerning the involvement of chickens PD-1/PD-L1 pathway in immunosuppression and tumor formation is required.

The increase in PD-1 expression was reported in the acute phase of HCV and lymphocytic choriomeningitis virus (LCMV) infection [26,27]. In chickens experimentally infected with MDV-1, the expression of *PD-1* mRNA was increased in the early cytolytic phase, but dramatically decreased following the onset of the latent infection (Figure 3A). We previously reported that MDV-1 induces apoptosis in CD4^+^ T cells during latent phase, and the number of CD4^+^ T cells is decreased [28]. These observations suggest that PD-1 may be expressed on the CD4^+^ T cells in the early cytolytic phase, and the expression of PD-1 may be decreased by apoptosis of CD4^+^ T cells before the onset of MDV latency. In contrast, the increase in *PD-L1* mRNA expression was observed in the latent phase (Figure 3B), suggesting that PD-L1 contribute to the establishment and/or maintenance of MDV-1 latency. Further studies, including the identification of PD-1- and PD-L1-expressing cell populations and the expression analysis of PD-1 and PD-L1 on the protein level, are needed to clarify the role of the PD-1/PD-L1 pathway in each phase of the infection.

In the case of HIV infection, some cytokines and viral factors are considered to be responsible for the increase in PD-1 and PD-L1 expressions [29], and it has been well characterized that PD-L1 expression could be regulated by IFN-γ [15]. In this study, the expression of *IFN-γ* mRNA was increased in the early cytolytic phase, and this expression kinetics was similar to that of *PD-1* mRNA (Figure 3C), suggesting that IFN-γ may be involved in the regulation of PD-1 expression. However, the increase in the *PD-1* mRNA expression was not observed in the secondary cytolytic phase despite the increase in the *IFN-γ* expression. Thus, PD-1 expression may be induced as a consequence of a negative feedback by IFN*-*γ stimulation during the acute phase. On the contrary, the expression of *PD-L*1 mRNA was transiently elevated at 7 d.p.i., and then, the expression of *IFN-γ* mRNA was increased (Figure 3B, C). At 21 d.p.i., however, *PD-L1* mRNA expression were increased again although *IFN-γ* mRNA expression was decreased (Figure 3B, C). The reason for these observations is unknown, but IFN-γ might gradually induce the expression of PD-L1, which, in turn, cause immunosuppression and reduce the IFN-γ expression.

PD-L1 is expressed on a variety of human and murine tumors [30], whereas tumor-infiltrating lymphocytes highly express PD-1 [31]. In the case of nodular lymphocyte-predominant Hodgkin lymphoma and angioimmunoblastic T-cell lymphoma, tumor cells express PD-1 [32,33]. Moreover, tumor cells caused by HTLV-1 express both PD-1 and PD-L1, and infiltrating T cells express PD-1, suggesting that PD-L1 expressed on these neoplastic CD4^+^ T cells induce immunosuppression of infiltrating T cells and contribute to the immune evasion [34]. In the case of MDV-1 infection, both *PD-1* and *PD-L1* mRNA expression were increased in tumor cells (Figure 4A, B, Figure 7A, B, Figure 8C, Additional file 1: Figure S1). Thus, both PD-1 and PD-L1 may be expressed on MD tumor cells, and may contribute to the immune evasion. Interestingly, the expression of *IFN-γ* mRNA was increased in tumor lesions (Figure 4)C, and the expression of *IFN-γ* mRNA in tumor lesions was higher than that in neighboring cells (data not shown). As a positive correlation was shown between *IFN-γ* and *PD-L1* mRNA expressions (Figure 5B), IFN-γ may upregulate the expression of *PD-L1* mRNA in tumor cells in an autocrine manner, and subsequently, PD-L1 may enhance the immunosuppression.

Several groups reported that viral proteins such as Nef protein of HIV and core protein of HCV cause the increase in PD-1 expression [23,24]. In this study, we focused on an MDV-1 oncoprotein, Meq, because Meq regulates the expressions of various genes as a transcription factor and is the most important viral factor related to MD pathogenesis [8,35]. The expressions of *meq* and *PD-1* mRNA were transiently increased at 14 d.p.i (Figure 3A, D), and in addition, the expression of *meq* mRNA was positively correlated with *PD-1* mRNA expression in tumor lesions (Figure 5A), indicating that Meq may regulate the expression of PD-1. However, a positive correlation between the expression of *meq* and *PD-L1* was not observed in the spleens and tumors from MDV-1-infected chickens. Since various viral factors are involved in MDV-1 oncogenesis and pathogenesis [35], other factors may correlate with the expressions of host immunoinhibitory molecules.

In summary, we demonstrated that host immunoinhibitory factors, PD-1 and PD-L1 were expressed in the spleens of MDV-1-infected chickens, and their expressions were showed different kinetics in each phase of the infection (Additional file 2: Figure S2). Furthermore, these factors were expressed in MD-derived tumors (Additional file 2: Figure S2). These results suggest that the PD-1/PD-L1 pathwayis involved in the immunosuppression and tumor formation by MDV-1.

## Materials & methods

### Cells

MD-derived lymphoblastoid cell lines, MSB1 [36] and HP1 [37], and chicken T-cell line, Lee1, that was transformed by REV and was established in our laboratory, were maintained at 41°C, 5% CO_2_ in RPMI 1640 (Sigma-Aldrich Co., St. Louis, USA) supplemented with 10% heat-inactivated fetal bovine serum (FBS; Invitrogen, Carlsbad, USA), 0.03% L-glutamine, 100 U/ml penicillin, 100 μg/ml streptomycin and 50 μM 2-mercaptoethanol. Chicken embryo fibroblasts (CEFs) were prepared from 11-day-old fertile eggs (Hokuren Co. Ltd, Sapporo, Japan) and maintained at 37°C, 5% CO_2_ in Eagle’s Minimum Essential Medium (Nissui, Tokyo, Japan) supplemented with 10% Tryptose phosphate broth (Difco Laboratories, Detroit, USA), 0.03%  L-glutamine, 100 U/ml penicillin, 100 μg/ml streptomycin and 0.1% NaHCO_3_. The immortalized CEF cell line, DF-1 [38], was maintained at 39°C, 5% CO_2_ in Dulbecco’s modified Eagle’s Medium (D-MEM; Invitrogen) supplemented with 10% FBS, 0.03% L-glutamine, 100 U/ml penicillin, 100 μg/ml streptomycin and 0.1% NaHCO_3_.

### Chickens

Neonatal male White Leghorn chickens were purchased from Hokuren Co. Ltd., and raised in isolators. The flock was free of common poultry diseases and not vaccinated against MDV. Feed and water were provided *ad libitum*.

### Virus

A strain of very virulent MDV-1, RB1B [39], was obtained from chicken kidney cell culture taken from experimentally infected chickens in our laboratory. This virus was propagated in CEFs and virus titer was determined by plaque assays as described previously [40]. These infected CEFs were used for the viral inoculation of chickens. Strain RB1B at passage 12 was used in this study.

### Virus inoculation and sample collection from experimentally infected chickens

Two groups of 5-day-old chickens (36 chickens/group) were inoculated intraperitoneally with either 2,000 plaque forming unit (PFU)/0.2 ml of RB1B or 0.2 ml of PBS as untreated controls. Spleens and PBMCs were collected from chickens in each group at 3, 7, 10, 14, 21, 28, and 35 d.p.i. Four samples per group were prepared at each time point. Each sample was a composite from two chickens at 3 d.p.i., because the number of cells collected from one chicken was too few to be examined. At 7 to 35 d.p.i. each sample was obtained from only one chicken. In addition, tumor lesions, which were observed in kidneys of MDV-1-infected chickens after 21 d.p.i., were also collected. This study was conducted in accordance with guidelines of the Institutional Animal Care and Use Committee of Hokkaido University, Japan.

### Samples of chickens with MD in the field

Samples of chickens with MD in the field were collected from poultry farms in Japan in 2010. Nine spleen- and 4 tumor-samples were used in this study. Of these tumor samples, one was observed in a spleen and the others were observed in livers.

### Evaluation of viral loads in MDV-1-infected chickens by real-time PCR

The absolute MDV-1 genome loads in the spleens and tumor lesions were quantified using real-time PCR with primers specific to the *meq* gene of MDV-1 as described elsewhere [41]. The sequences of the *meq* primers are shown in Table 1. Total cellular DNA was extracted using the Sepa Gene kit (Sanko Jyunyaku Co. Ltd., Tokyo, Japan) according to the manufacturer’s instructions. Real-time PCR assays were performed using SYBR Premix DimerEraser (Takara, Shiga, Japan) and a LightCycler 480 System II (Roche Diagnostics, Mannheim, Germany). The chicken *β-actin* gene in each sample was also amplified using a primer pair in Table 1. The *β-actin* gene was used as a reference for the *meq* gene in each sample to express the ratio between the two genes. Each sample was tested in duplicate and the data were presented as average.

**Table 1:** Primers used for real-time PCR, real-time RT-PCR, and LMD and RT-PCR

**Genes**	**Analysis**	**Types**	**Sequences**	**Annealing temperature for LMD and RT-PCR analysis**	**Accession number**
*meq*	real-time PCR	Forward	5′-GTCCCCCCTCGATCTTTCTC-3′	60°C	AY362736
	real-time RT-PCR	Reverse	5′-CGTCTGCTTCCTGCGTCTTC-3′		
	LMD and RT-PCR				
*β-actin*	real-time PCR	Forward	5′-GAGAAATTGTGCGTGACATCA-3′	55°C	L08165
		Reverse	5′-CCTGAACCTCTCATTGCCA-3′		
*β-actin*	real-time RT-PCR	Forward	5′-CCAACTGGGATGATATGGAGAAG-3′	58°C	NM205518
	LMD and RT-PCR	Reverse	5′-AGGCATACAGGGACAGCACA-3′		
*PD-1*	real-time RT-PCR	Forward	5′-GGACTACGGTGTGCTGGAGTT-3′	60°C	XM422723
	LMD and nested RT-PCR (2nd PCR)	Reverse	5′-TCTTTCCTCGCTCTGGTGTG-3′		
*PD-1*	LMD and nested RT-PCR (1st PCR)	Forward	5′-ACACCCTGGCGGAGGTGAAG-3′	60°C	XM422723
(1st PCR)		Reverse	5′-TGCCAGGTCCTCTGATCGTTGTG-3′		
*PD-L1*	real-time RT-PCR	Forward	5′-TTCAGGGACGGATAAAGCTG-3′	60°C	XM424811
	LMD and nested RT-PCR (2nd PCR)	Reverse	5′-CGTCTCTGAGCTTCACGTTG-3′		
*PD-L1*	LMD and nested RT-PCR (1st PCR)	Forward	5′-GGGACTTAAGCGTCATCTGGGAA-3′	58°C	XM424811
(1st PCR)		Reverse	5′-GGTAAAGCCCCGCGTCTCTG-3′		
*IFN-γ*	real-time RT-PCR	Forward	5′-CTCCCGATGAACGACTTGAG-3′	58°C	NM205149
		Reverse	5′-CTGAGACTGGCTCCTTTTCC-3′		

### Total cellular RNA extraction and cDNA synthesis

Total cellular RNA was extracted from MSB1, HP1, and Lee1 cells, PBMCs obtained from a healthy chicken, and the spleen and tumor samples from experimentally infected chickens and from chickens with MD in the field, using the TRIZOL reagent (Invitrogen) according to the manufacturer’s protocol. Each RNA sample was treated with DNase I (Invitrogen) to remove residual DNA, and cDNA was synthesized with Moloney murine leukemia virus reverse transcriptase (Takara) as directed by the manufacturer.

### Expression analyses of the *PD-1*, *PD-L1*, *IFN-γ*, and *meq* mRNA by real-time RT-PCR

Synthesized cDNA samples were used to determine the mRNA expression levels of related factors by real-time RT-PCR. The cDNA template was added to a total volume of 20 μl containing PCR buffer, oligonucleotide primers at 0.3 μM each of primer, and 10 μl of SYBR Premix DimerEraser. Real-time RT-PCR assays were performed using the LightCycler 480 System II (Roche Diagnostics). The sequences of specific primers and accession numbers for *PD-1*, *PD-L1*, and *IFN-γ* are listed in Table 1, and *meq* as described above. The cycling condition consists of initial template denaturing at 95°C for 30 s, followed by amplification of template for 35 cycles (*β-actin*) or 40 cycles (*PD-1*, *PD-L1*, *IFN-γ*, and *meq*) of 95°C 5 s, 55°C for 30 s, and 72°C for 30 s. A final melting curve analysis was performed from 65°C to 95°C at a rate of 0.11°C/s (continuous acquisition), with a final cooling to 40°C over 10 s. The specificity of amplification was confirmed by melting point analysis. The chicken *β-actin* gene in each sample was also amplified using a primer pair in Table 1. The *β-actin* gene was used as reference for target genes in each sample to express the ratio between the two genes. Serial dilutions of pGEM-T easy vector (Promega, Madison, WI, USA) encoding each gene (1 × 10^−6^ to 1 ng/ml) were used to generate standard curves for quantification. All primers were BLAST-searched against chicken DNA sequences available in GenBank to ensure amplification specificity and synthesized by Hokkaido System Science (Sapporo, Japan). All samples were tested in duplicate and the data were presented as average. Results were expressed in folds of each mRNA expression compared to those in PBMCs or spleens of uninfected chickens.

### *in situ* hybridization analysis

cRNA probes for the *meq* gene were synthesized in the presence of digoxigenin-labbeled UTP by using the DIG RNA labeling kit (Roche Diagnostics) according to the manufacturer’s instructions. The primers used for making each probe were M-S (5′-ATGTCTCAGGAGCCAGAGCCGGGCGCT-3′) and M-AS (5′-GGGGCATAGACGATGTGCTGCTGAG-3′) as described previously [42]. The RNA probes (designated *meq*-sense and *meq*-antisense) were stored at −80°C until use. The kidney sample including tumor lesions and spleen samples were fixed in 4% paraformaldehyde (PFA) overnight at 4°C and paraffin sections (0.4 μm thick) were then prepared. Deparaffinized, proteinase K-digested sections were incubated with a prehybridization solution and then incubated with hybridization buffer containing 50% formamide, 10 mM Tris–HCl pH 7.4, 200 μg/ml tRNA, 1 × Denhardt’s solution (0.02% bovine serum albumin, 0.02% polyvinylpyrrolidone, and 0.02% Ficoll PM400 (Amersham Pharmacia, Uppsala, Sweden)), 10% dextran sulphate, 0.25% sodium dodecyl sulphate, 1 mM ethylenediamine tetraacetic acid pH 8.0, and 50 ng of sense or antisense RNA probe overnight at 58°C. The sections were incubated in an anti-DIG conjugated to alkaline phosphatase (1:400; Roche Diagnostics) overnight at room temperature (RT). The signal was detected by incubation of the sections with substrate solution containing nitroblue tetrazolium/ X-phosphate in a solution composed of 100 mM Tris–HCl (pH 9.5), 100 mM NaCl, and 50 mM MgCl_2_ in a dark room overnight at RT.

### RT-PCR or nested RT-PCR assay of laser-captured microdissections

The organs were fixed with 4% PFA and embedded to paraffin for laser microdissection (LMD). LMD was performed as previously reported [43]. First, 5 μm-thick paraffin sections were mounted on glass slides precoated with LMD films (Meiwafosis, Tokyo, Japan), deparaffinized by xylene, and dehydrated by alcohol. After staining with 1% toluidine blue for 5 sec, LMD was performed on the normal renal cortices and tumorigenic lesions by using Ls-Pro300 (Meiwafosis), according to the manufacturer’s protocol. All procedures were performed in RNase-free conditions.

Total RNA purified with RNAqueous (Ambion, Austin, TX, USA) was reverse-transcribed to cDNA by using SuperScript First-Strand Synthesis System for RT-PCR (Invitrogen) according to the manufacturer’s protocol. Synthesized cDNA was used for the PCR or nested PCR with TAKARA-Taq (Takara) and appropriate primer pairs (Table 1). Reactions were started with 94°C for 5 min, followed by 40 cycles of 94°C for 30 sec, each annealing temperature (shown in Table 1) for 30 sec and 72°C for 30 sec, and f 72°C for 7 min, and finally kept at 4°C. Nested PCR analysis was performed by using 1 μl of the 1st PCR reaction as a template and in a 20 μl reaction mixture. The amplified fragments were separated on agarose gels (2.0%) and visualized under ultraviolet light after staining with ethidium bromide.

### Analysis of the immunoinhibitory function in the PD-1/PD-L pathway

For the construction of plasmids expressing PD-L1, chicken *PD-L1* transcript was amplified by PCR using primes PDL1-F-*Not*I (5′-GGGGCGGCCGCATGATGGAAAAGCTTTTGCTTTTGCAC-3′) and PDL1-R-*Sal*I (5′-CCCGTCGACTTTATGCTTACATTTCAGCTCCGCATCTT-3′) that added a *Not*I site to the 5′ end and a *Sal*I site to the 3′ end for cloning. The amplified fragment was digested with *Not*I and *Sal*I and cloned into the *Not*I and *Sal*I sites of the pCMV-Tag1 vector (Stratagene, La Jolla, USA) to construct a plasmid expressing PD-L1 (pCMV-PDL1).

DF-1 cells were seeded in 6-well plate at 2 × 10^6^ cells per well in 2.5 ml of D-MEM and incubated at 41°C in 5% CO_2_ overnight. The cells in each well were transfected with 5 μg of pCMV-PDL1 or pCMV-Tag1 using Lipofectamine 2000 (Invitrogen) according to the manufacturer’s instructions. At 36 h post transfection, these DF-1 cells were co-cultured with Lee1 cells at 8 × 10^6^ cells per well in 2.5 ml of RPMI 1640 and incubated at 41°C in 5% CO_2_ for 12 h. The expression levels of the *IFN-γ* gene in co-cultured Lee1 cells were analyzed by real-time RT-PCR as described above.

### Western blotting

Transfected DF-1 cells were lysed at 48 h post transfection in 2 × SDS buffer (150 mM Tris–HCl pH 6.8, 4% SDS, 10% 2-mercaptoethanol, 20% glycerol and 0.2% bromophenol blue) and boiled for 10 min. Samples were separated on 15% SDS-polyacrylamide gels and transferred to the polyvinylidene difluoride membranes (Millipore Corp., Bedford, MA, USA). The membranes were blocked overnight at 4°C with 0.05% Tween 20 in phosphate-buffered saline (PBST) containing 3% skim milk. The membranes were then incubated at RT for 1 h with goat anti-myc tag antibody (abcam), washed 3 times with PBST, and incubated at RT for 30 min with peroxidase-conjugated rabbit anti-goat IgG (EY Laboratories). After 3 washes with PBST, the membranes were incubated with 3,3′-diaminobenzidine tetrahydrochloride and cobalt chloride substrates to visualize the peroxidase signal. The blot was also probed with mouse anti-actin monoclonal antibody (Millipore) followed by peroxidase-conjugated goat anti-mouse IgG (H + L) (Jackson ImmunoRsearch) as a loading and transfer control.

## Abbreviations

PD-1, programmed death-1; PD-L, programmed death ligand; MDV, Marek’s disease virus; IFN, Interferon; RT-PCR, reverse transcription polymerase chain reaction; HVT, herpesvirus of turkeys; TCR, T cell receptor; HIV, human immunodeficiency virus; HCV, hepatitis C virus; DCs, dendritic cells; HTLV-1, human T-cell lymphotropic virus type 1; PBMC, peripheral blood mononuclear cell; REV, reticuloendotheliosis virus; LCMV, lymphocytic choriomeningitis virus; RT, room temperature; CEF, chicken embryo fibroblast; LMD, laser microdissection.

## Competing interests

The authors declare that they have no competing interests.

## Authors’ contributions

AMK and SM carried out study design, most of the experiments, wrote the manuscript, and performed the statistical analysis. MI, RK, ST, and OI helped in vitro experiments. SK, and KO revised the manuscript. All authors read and approved the final manuscript.

## Supplementary Material

Additional file 1 **Figure S1.** Comparison of tumor lesions observed in kidneys with PBMCs obtained from infected or uninfected chickens. A total of 9 tumor samples and PBMCs obtained from tumor-bearing chickens were collected at 21, 28, and 35 d.p.i. (*n* = 2, 3, and 4, respectively). Twelve PBMCs obtained from uninfected chickens at 21, 28, and 35 d.p.i. The expressions of *PD-1* (A) and *PD-L1* (B) mRNA were determined by real-time RT-PCR. The concentration of each mRNA was normalized to that of *β-actin* mRNA. Error bars represent standard deviations. The significant differences were determined by Student’s *t*-test (*P < 0.05, **P < 0.01). Click here for file

Additional file 2 **Figure S2.** Hypothetical model of PD-1 and PD-L1 involvement in chickens infected with MDV-1. The expression of PD-1 is increased in the early cytolytic phase of the MDV infection, and may be involved in MD pathogenesis including apoptosis of CD4^+^T cells. In contrast, the expression of PD-L1 is increased in the latent phase, and may contribute to the establishment and maintenance of MDV-1 latency. Both PD-1 and PD-L1 are expressed on MD tumor cells in the secondary cytolytic phase, and thereby may contribute to the immunosuppression, immune evasion, and tumor development. Click here for file
