# Supplementary figures and images for: Molecular characterization of immunoinhibitory factors PD-1/PD-L1 in chickens infected with Marek’s disease virus
Source: Virol J. 2012 May 21;9:94. doi: 10.1186/1743-422X-9-94 (PMC3447683; doi:10.1186/1743-422X-9-94)

A

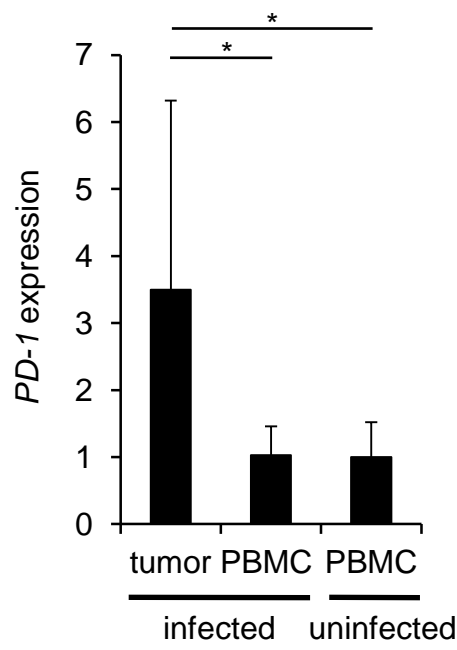

B

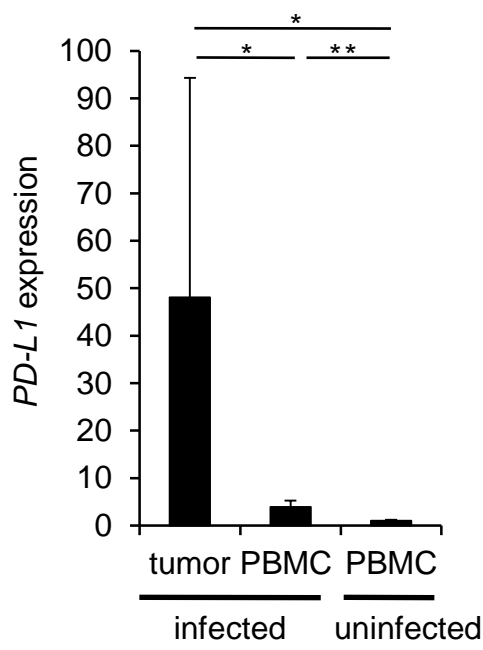

Supplement: Additional file 1 — Figure S1. Comparison of tumor lesions observed in kidneys with PBMCs obtained from infected or uninfected chickens. A total of 9 tumor samples and PBMCs obtained from tumor-bearing chickens were collected at 21, 28, and 35 d.p.i. (n = 2, 3, and 4, respectively). Twelve PBMCs obtained from uninfected chickens at 21, 28, and 35 d.p.i. The expressions of PD-1 (A) and PD-L1 (B) mRNA were determined by real-time RT-PCR. The concentration of each mRNA was normalized to that of β-actin mRNA. Error bars represent standard deviations. The significant differences were determined by Student’s t-test (*P < 0.05, **P < 0.01). [file 1743-422X-9-94-S1.pdf]
