# Supplementary material for: Molecular characterization of immunoinhibitory factors PD-1/PD-L1 in chickens infected with Marek’s disease virus
Source: Virol J. 2012 May 21;9:94. doi: 10.1186/1743-422X-9-94 (PMC3447683; doi:10.1186/1743-422X-9-94)
Supplement: Additional file 2 — Figure S2. Hypothetical model of PD-1 and PD-L1 involvement in chickens infected with MDV-1. The expression of PD-1 is increased in the early cytolytic phase of the MDV infection, and may be involved in MD pathogenesis including apoptosis of CD4+T cells. In contrast, the expression of PD-L1 is increased in the latent phase, and may contribute to the establishment and maintenance of MDV-1 latency. Both PD-1 and PD-L1 are expressed on MD tumor cells in the secondary cytolytic phase, and thereby may contribute to the immunosuppression, immune evasion, and tumor development. [file 1743-422X-9-94-S2.pdf]

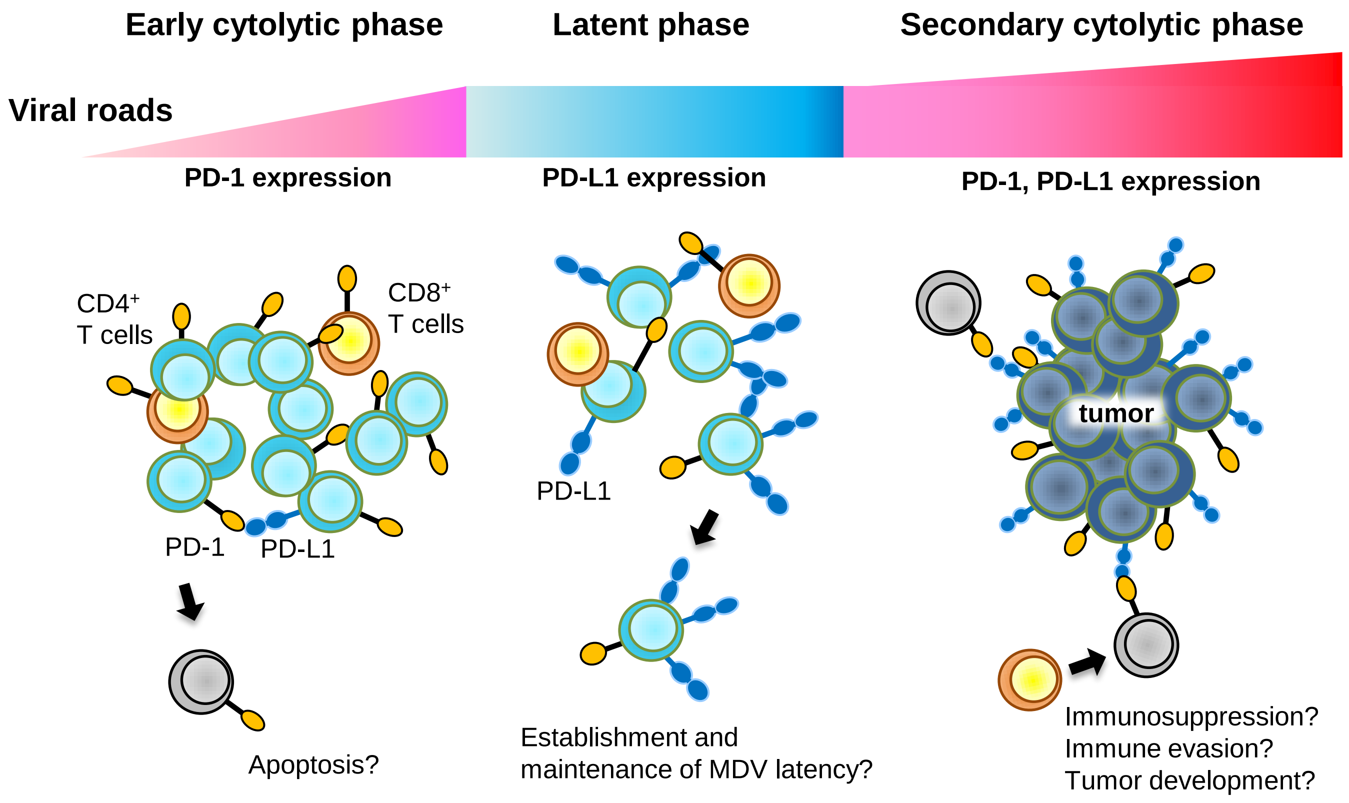

Early cytolytic phase
Latent phase
Secondary cytolytic phase
Viral roads
PD-1 expression
PD-L1 expression
PD-1, PD-L1 expression
CD4+ T cells
CD8+ T cells
PD-1
PD-L1
Apoptosis?
PD-L1
Establishment and maintenance of MDV latency?
tumor
Immunosuppression?
Immune evasion?
Tumor development?
